# Plant-Derived Polyphenols Interact with Staphylococcal Enterotoxin A and Inhibit Toxin Activity

**DOI:** 10.1371/journal.pone.0157082

**Published:** 2016-06-07

**Authors:** Yuko Shimamura, Natsumi Aoki, Yuka Sugiyama, Takashi Tanaka, Masatsune Murata, Shuichi Masuda

**Affiliations:** 1 School of Food and Nutritional Sciences, University of Shizuoka, 52–1 Yada, Suruga-ku, Shizuoka 422–8526, Japan; 2 Graduate School of Biochemical Science, Nagasaki University, 1–14 Bukyo-machi, Nagasaki 852–8521, Japan; 3 Department of Nutrition and Food Science, Ochanomizu University, 2-1-1 Otsuka, Bunkyo-ku, Tokyo 112–8610, Japan; Wageningen University, NETHERLANDS

## Abstract

This study was performed to investigate the inhibitory effects of 16 different plant-derived polyphenols on the toxicity of staphylococcal enterotoxin A (SEA). Plant-derived polyphenols were incubated with the cultured *Staphylococcus aureus* C-29 to investigate the effects of these samples on SEA produced from C-29 using Western blot analysis. Twelve polyphenols (0.1–0.5 mg/mL) inhibited the interaction between the anti-SEA antibody and SEA. We examined whether the polyphenols could directly interact with SEA after incubation of these test samples with SEA. As a result, 8 polyphenols (0.25 mg/mL) significantly decreased SEA protein levels. In addition, the polyphenols that interacted with SEA inactivated the toxin activity of splenocyte proliferation induced by SEA. Polyphenols that exerted inhibitory effects on SEA toxic activity had a tendency to interact with SEA. In particular, polyphenol compounds with 1 or 2 hexahydroxydiphenoyl groups and/or a galloyl group, such as eugeniin, castalagin, punicalagin, pedunculagin, corilagin and geraniin, strongly interacted with SEA and inhibited toxin activity at a low concentration. These polyphenols may be used to prevent *S*. *aureus* infection and staphylococcal food poisoning.

## Introduction

Staphylococcal food poisoning is caused by staphylococcal enterotoxins (SEs) produced by *Staphylococcus aureus* [1]. *S*. *aureus* produces a group of 11 SEs, including staphylococcal enterotoxin A (SEA), and 11 SE-like toxins [2]. Among them, SEA is the most closely connected with staphylococcal food poisoning [3]. Only 144 ng of SEA in chocolate milk was sufficient to cause human food poisoning [4], and a similar amount of SEA in skim milk powder affected over 14,000 people [5]. In Japan, foods implicated in staphylococcal food poisoning include rice balls, omelettes in lunch boxes, milk and yogurt drinks [5]. Therefore, novel strategies for neutralizing SEA toxin activity or inhibiting SEA production are needed.

Plant-derived polyphenols are naturally occurring compounds that are largely found in fruits, vegetables, cereals and beverages. Fruits, such as apples, grapes and berries, contain up to 200–300 mg polyphenols per 100 g fresh weight [6]. Plant-derived polyphenols are thought to exert anti-viral and anti-bacterial effects [7, 8]. Apple juice, apple polyphenols and 4-hydroxytyrosol, an olive compound, inhibited splenocyte proliferation by inducing SEA [9, 10]. It is believed that the mechanism of detoxification of SEA by these compounds depends on the inhibition of the *in vivo* molecular interactions between the SEs and toxin receptor sites [11]. Our previous study demonstrated that tea polyphenols could inhibit the production or toxin activity of SEA [12]. However, the relationship between the polyphenol structure and the inhibition of staphylococcal toxin activity remains unknown. Although tannins have long been used in the food industry and manufacturing, there are no data available on the effects of plant-derived polyphenols, particularly tannins, in the production and toxin activity of SEA.

In the present study, we investigated whether 16 different plant-derived polyphenols interact with SEA at concentrations that do not inhibit *S*. *aureus* growth and examined the inhibitory effect of these polyphenols on SEA toxin activity. Additionally, the relationship between the polyphenol structure and the inhibition of toxin activity was determined.

## Materials and Methods

### Bacterial culture

The culture method for *S*. *aureus* C-29 (a SEA-producing strain) has previously been described in detail [13, 14]. Briefly, bacterial culture (3 mL) was added to the brain–heart infusion (BHI; Oxoid Ltd., Ogdensburg, NY, USA) broth, followed by incubation at 37°C with shaking for 16–18 h. The minimum inhibitory concentration (MIC) of polyphenols against *S*. *aureus* C-29 was measured using broth micro-dilution assays, as previously described [12].

### Chemicals

Sixteen different plant-derived polyphenols, including twelve hydrolysable tannins and four procyanidins, were used. Tannic acid (polygalloyl-glucose from the gall nuts of *Rhus semialata*) was purchased from Wako Pure Chemical Industries, Ltd. (Osaka, Japan). Other polyphenols used in the present study were obtained from our previous studies. 1-*O*-Galloyl-β-D-glucose (β-glucogallin, ≥95% purity; PubChem CID: 124021), 1,3,6-tri-*O*-galloyl-β-D-glucose (tri-galloyl-glucose, ≥90% purity; PubChem CID: 54125251) and 1,2,3,4,6-penta-*O*-galloyl-β-D-glucose (penta-galloyl-glucose, ≥95% purity; PubChem CID: 16177653) were isolated from the fruits of *Paeonia lactiflora* [15]. 1,3,4,6-Tetra-*O*-galloyl-β-D-glucose (tetra-galloyl-glucose, ≥90% purity; PubChem CID: 54091010) was obtained from the leaves of *Castanopsis fissa* [16]. Persimmon tannin oligomer (mixture mainly consisting of tetramer) was obtained from persimmon fruits [17]. Corilagin (≥95% purity; PubChem CID: 73568) and geraniin (≥90% purity; PubChem CID: 3001497) were isolated from the leaves of *Elaeocarpus sylvestris* var. *ellipticus* [18]. Sanguiin H-6 (≥90% purity; PubChem CID: 16131123), eugeniin (≥95% purity; PubChem CID: 442679) and pedunculagin (≥90% purity; PubChem CID: 442688) were isolated from the underground portion of *Sanguisorba officinalis* [19]. Castalagin (≥95% purity; PubChem CID: 168165) was obtained from the wood of *Castanea crenata* [20]. Punicalagin (≥90% purity; PubChem CID: 44584733) was isolated from the bark of a pomegranate tree [21]. Procyanidins B1 (≥90% purity; PubChem CID: 11250133) and B2 (≥85% purity; PubChem CID: 122738) as well as a mixture of procyanidin oligomer (blueberry) were obtained from the leaves of a rabbiteye blueberry [22].

### Interactions between polyphenols and cultured SEA-producing strains

The concentration of each polyphenol sample was the MIC or lower. The interactions between 16 plant-derived polyphenols and cultivated SEA-producing strains were estimated as previously described [12]. Briefly, 180 μL of bacterial suspension (10^3^–10^4^ CFU/mL) were mixed with 20 μL of each polyphenol at various concentrations (final concentration 0.1–0.5 mg/mL) and incubated at 37°C for 24 h. After incubation, the supernatants were collected and evaluated with SDS-PAGE and Western blot analysis.

### Interactions of SEA and polyphenols

The interactions of SEA and plant-derived polyphenols were investigated as previously described [12]. Briefly, 10 μL of 100 ng/mL purified SEA (Toxin Technology, Sarasota, FL, USA) were mixed with 90 μL of various concentrations of polyphenol test samples (final concentration 0.1–0.25 mg/mL) and incubated at 37°C for 24 h. After incubation, the supernatants were collected and evaluated with SDS-PAGE and Western blot analysis.

### SDS-PAGE and Western blot analysis

SDS-PAGE and Western blot analysis were performed as previously reported with some modifications [23]. Briefly, equal amounts of the supernatants and the sample buffer (250 mM Tris, pH 7, 4% SDS, 20% glycerol, 10% β-mercaptoethanol and 0.05% bromophenol blue; Wako Pure Chemical Industries, Ltd., Osaka, Japan) were treated at 110°C for 5 min. Then, 15 μL aliquots were evaluated with SDS–PAGE on a 15% sodium dodecyl sulfate (SDS)-polyacrylamide gel. After electrophoresis, the protein bands were transferred to a polyvinylidene difluoride membrane. The membranes were probed with 1:20,000 (v/v) of rabbit anti-SEA IgG (Sigma, St. Louis, MO, USA, Lot Number 103K4831) and then reacted with 1:500 (v/v) of goat anti-rabbit peroxidase (KPL). SEA combined with the membrane was evaluated using a Protein Detector Western Blot Kit BCIP/NBT System (KPL, Gaithersburg, MD, USA) according to the manufacturer’s instructions. The results were quantified with Image J software (National Institutes of Health, Bethesda, MD, USA).

### Preparation of mouse spleen cells

The preparation of mouse spleen cells (isolation) was conducted as previously described [12]. Spleen cells were collected with aseptic manipulation from C57BL/6 female mice (Japan SLC, Shizuoka, Japan) with disposable homogenizers (BioMasher II, Nippi. Japan) in a Russ-10 cell culture medium. The suspension of cells was then filtered through a 40 μm nylon cell strainer (BD Falcon, BD Biosciences, San Jose, CA, USA). After erythrocyte lysis with ACK Lysing Buffer (Gibco), the cells were resuspended in the Russ-10 medium. The cells were then prepared at a concentration of 1 × 10^6^ cells/mL. All experimental animal procedures were performed with the approval of the Institutional Animal Care and Use Committee of the University of Shizuoka (Permit Number: 135018 and 145058) based on those of the American Association for Laboratory Animal Science. Mice were sacrificed with sodium pentobarbital anesthesia and killed by rapid neck disarticulation. All efforts were made to minimize animals suffering. Twelve mice were used.

### SEA activity assays

The inhibitory effects of polyphenols on SEA activity, using the assay for an enzymatic cleavage, was estimated with the MultiTox-Fluor Multiplex Cytotoxicity Assay (Promega Co., Madison, WI, USA) according to the manufacturer’s instructions [12]. Spleen cells were plated in individual wells in 96-well plates (2.5 X 10^5^/mL) in a Russ-10 medium and treated with SEA (200 ng/mL) and polyphenol samples (1–25 μg/mL), followed by incubation at 37°C in a 5% CO_2_ incubator. After incubation for 48 h, the fluorescent emission intensity produced after the cleavage reaction of the glycyl-phenylalanyl-aminofluorocoumarin (GF-AFC) from viable cells (excitation at 355 nm and emission at 523 nm) was determined.

### The theoretical relationship between the polyphenol structure and the inhibition of staphylococcal toxin activity

Polyphenols that had inhibitory effects on SEA toxic activity had a tendency to interact with SEA. Using western blot analysis, we examined whether the SEA protein band intensity after treatment with polyphenols was correlated with the polyphenol structure and inhibition of staphylococcal toxin activity. To examine the theoretical relationship between the SEA protein band intensity and the treatment with polyphenols with consideration of the western blot analysis and polyphenol structure, an affinity interaction unit (AIU) of polyphenols and SEA was defined by the following equation:

$$AIU\;=\;100-\left\{\frac{(Band\;intensity\;of\;SEA\;treated\;with\;a\;polyphenol)\times 100}{Band\;intensity\;of\;SEA\;(\text{control})}\right\}$$

### Statistical analysis

Each experiment was performed at least three times. The results are given as the means ± SD. All data were analyzed by one-way ANOVA, which was followed by the Dunnett’s test. The level of significance was set at *p* < 0.05 or 0.01. AIU values were calculated based on the degree of the band intensity of SEA by Western blot analysis. The band intensity of SEA treated with each polyphenol was analyzed using 1-factor ANOVA, and differences between individual group means were analyzed using the Tukey-Kramer test.

## Results and Discussion

### Determination of the MIC

**Fig 1:**
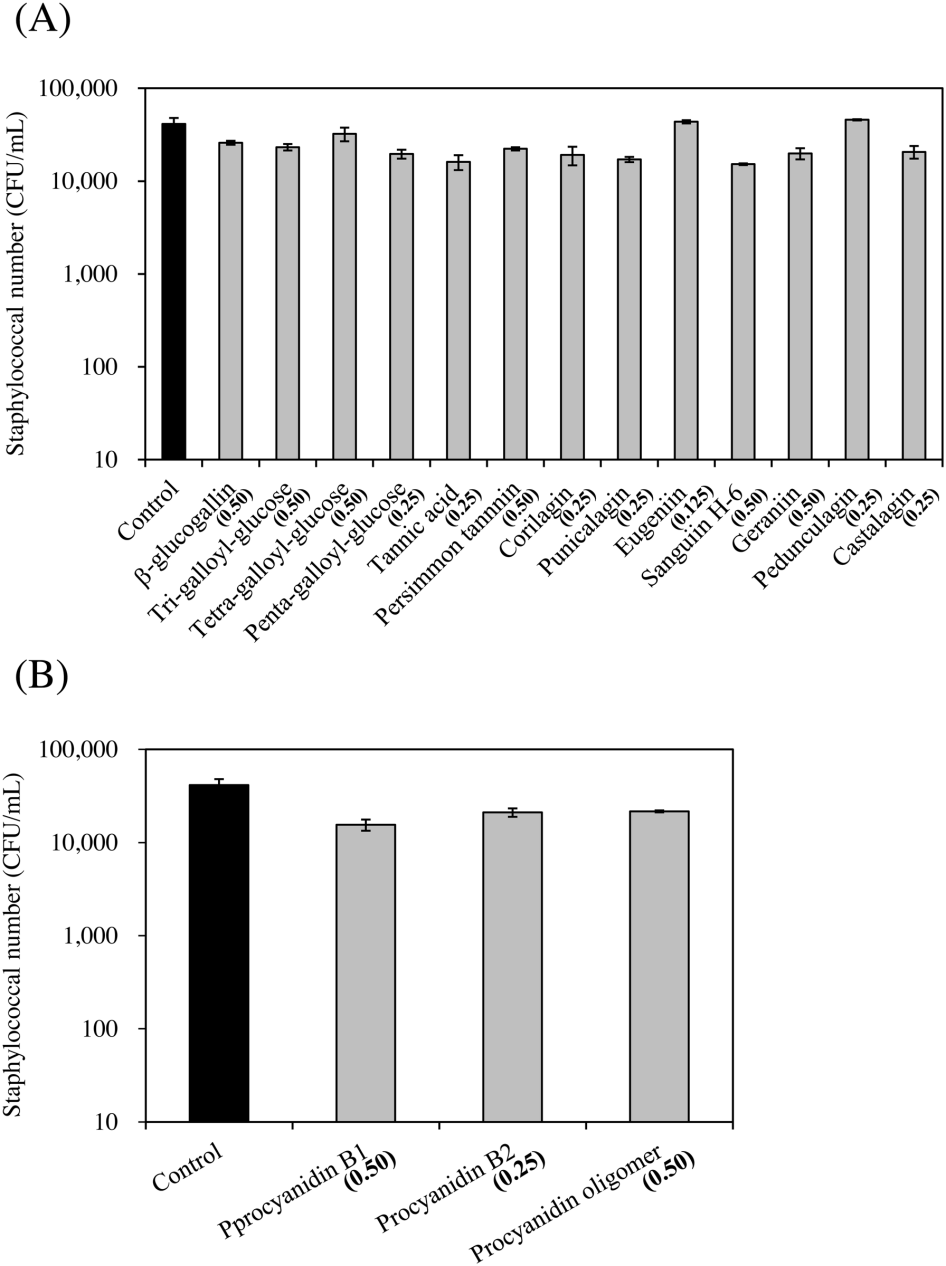
Growth effect of polyphenols on SEA-producing strain. (A) hydrolyzable tannins and (B) procyanidins. Values represent the mean ± SD for three independent experiments. The final concentration of polyphenol (mg/mL) is indicated between brackets.

We confirmed that the interaction of polyphenols with the cultured SEA-producing strain did not affect the staphylococcal growth. The MIC values when *S*. *aureus* C-29 were treated with polyphenols were determined. Only eugeniin exhibited strong anti-bacterial activity (MIC: 0.125 mg/mL). β-glucogallin, tri-galloyl-glucose, tetra-galloyl-glucose, persimmon tannin, sanguiin H-6, geraniin, procyanidin B_1_ and procyanidin oligomer exhibited weak anti-bacterial activity (MIC > 0.50 mg/mL). Penta-galloyl-glucose, tannic acid, corilagin, punicalagin, pedunculagin, castalagin and procyanidin B2 had more antibacterial activity than the above polyphenols (MIC: 0.25 mg/mL) (Table 1). In general, *S*. *aureus* (a Gram-positive bacterium) is known for its high sensitivity to phenolic extracts [24]. Taguri et al. reported that 10 different plant polyphenols, including tannic acid, punicalagin, castalagin and geraniin, inhibit the growth of *S*. *aureus*. The range of MIC values for each plant polyphenol against *S*. *aureus* was 0.098–0.389 mg/mL. It has already been reported that polyphenols with pyrogallol groups have strong anti-bacterial activities, and those with catechol and resorcinol rings have lower anti-bacterial activity [8, 24]. Our results of the MIC of each plant polyphenol against *S*. *aureus* (0.125–0.50 mg/mL) was similar to previous reports [8, 24]. To screen the polyphenols specifically affecting SEA production without inhibiting staphylococcal growth, we investigated whether the compounds would affect the growth of a cultured SEA-producing strain (Fig 1). *S*. *aureus* was grown at 0.5 mg/mL for almost all polyphenols. Therefore, a 0.5 mg/mL solution of each polyphenol was diluted to 1/2 of the original concentration. All subsequent assays were conducted at a concentration that did not inhibit the growth of *S*. *aureus* (0.125, 0.25 and 0.50 mg/mL; lower than each MIC value).

**Table 1:** Mean minimum inhibitory concentration (MIC (mg/mL)) in test samples against *Staphylococcus aureus* C-29.

Samples	MIC (mg/mL)
β-glucogallin	>0.50
Tri-galloyl-glucose	>0.50
Tetra-galloyl-glucose	>0.50
Penta-galloyl-glucose	0.50
Tannic acid	0.50
Persimmon tannnin oligomer	>0.50
Corilagin	0.50
Punicalagin	0.50
Eugeniin	0.25
Sanguiin H-6	>0.50
Geraniin	>0.50
Pedunculagin	0.50
Castalagin	0.50
Procyanidin B1	>0.50
Procyanidin B2	0.50
Procyanidin oligomer	>0.50

β-glucogallin; 1-*O*-galloyl-β-D-glucose, tri-galloyl-glucose; 1,3,6-tri-*O*-galloyl-β-D-glucose, tetra-galloyl-glucose; 1,3,4,6-tetra-*O*-galloyl-β-D-glucose, penta-galloyl-glucose; 1,2,3,4,6-penta-*O*-galloyl-β-D-glucose, tannic acid; poly-galloyl-glucose.

### Interaction between polyphenols and the cultured SEA-producing strain

**Fig 2:**
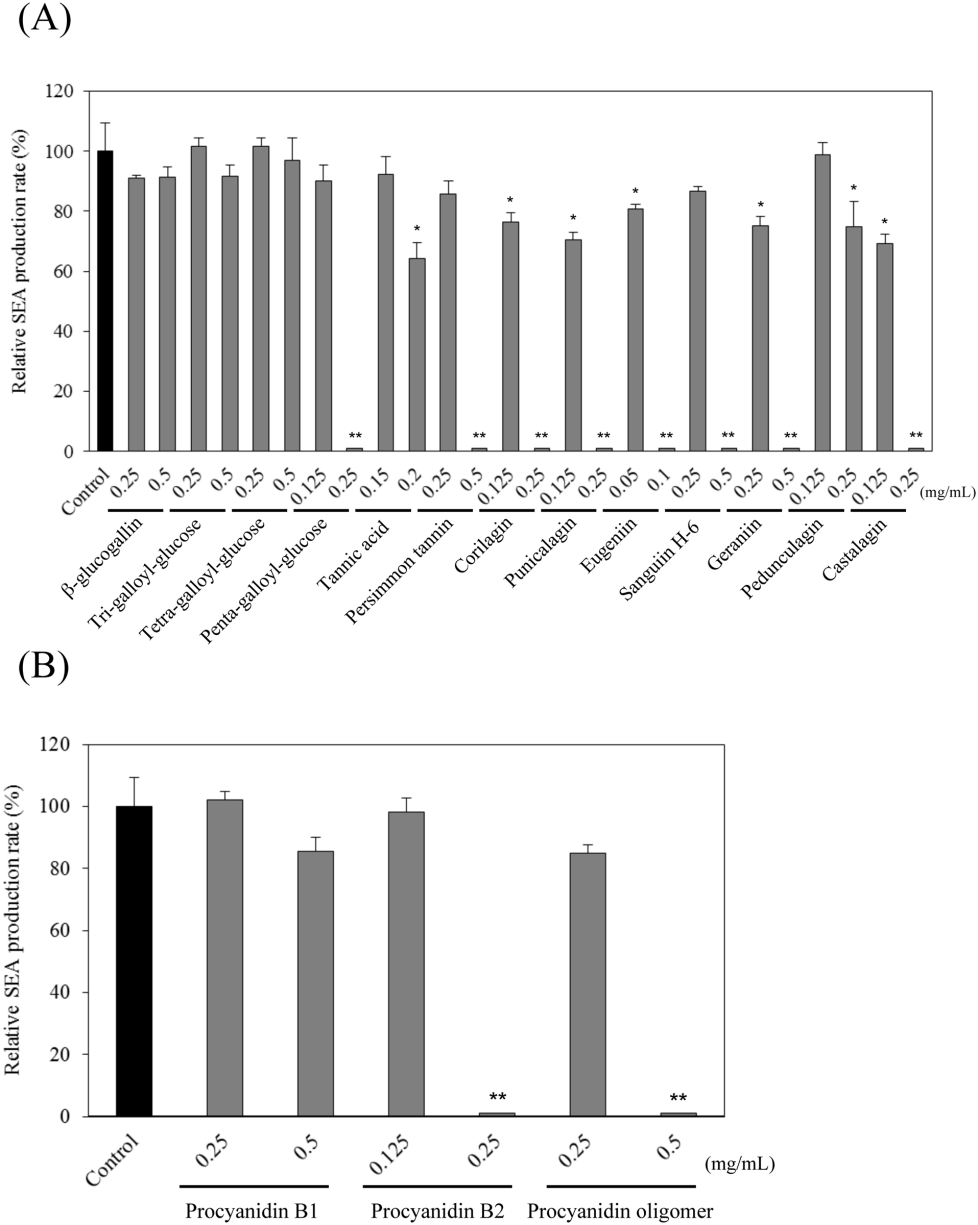
Western blot analysis of the influence of polyphenols on SEA production. (A) hydrolyzable tannins and (B) procyanidins. Values represent the mean ± SD for three independent experiments. * represents *p* < 0.05 compared to control, and ** represents *p* < 0.01 compared to control.

The interaction between 16 plant-derived polyphenols and *S*. *aureus* C-29 was investigated. As a result, 10 polyphenols significantly down-regulated the 27 kDa SEA protein band by Western blot analysis. At concentrations of 0.5 mg/mL of persimmon tannin, sanguiin H-6, geraniin and procyanidin oligomer; 0.25 mg/mL of penta-galloyl-glucose, corilagin, punicalagin, castalagin and procyanidin B2; and 0.1 mg/mL of eugeniin, SEA protein was not detected in the Western blot analysis (*p* < 0.01) (Fig 2(A) and 2(B)). However, the band intensity of the SEA protein was not entirely suppressed by the following 2 polyphenols: tannic acid (0.2 mg/mL) and pedunculagin (0.25 mg/mL); SEA protein signal diminished (*p* < 0.05) in a dose-dependent manner (Fig 2(A)). Four polyphenols (β-glucogallin, tri-galloyl-glucose, tetra-galloyl-glucose (Fig 2(A) and procyanidin B1 (Fig 2(B)) did not inhibit the SEA protein signal compared with the untreated cultured SEA-producing strain (control). These results suggested that 12 polyphenols (10 with an absent and 2 with a diminished SEA protein band) directly reacted with SEA, inhibited the production of SEA, or inhibited SEA gene expression.

### Interaction of SEA and polyphenols

**Fig 3:**
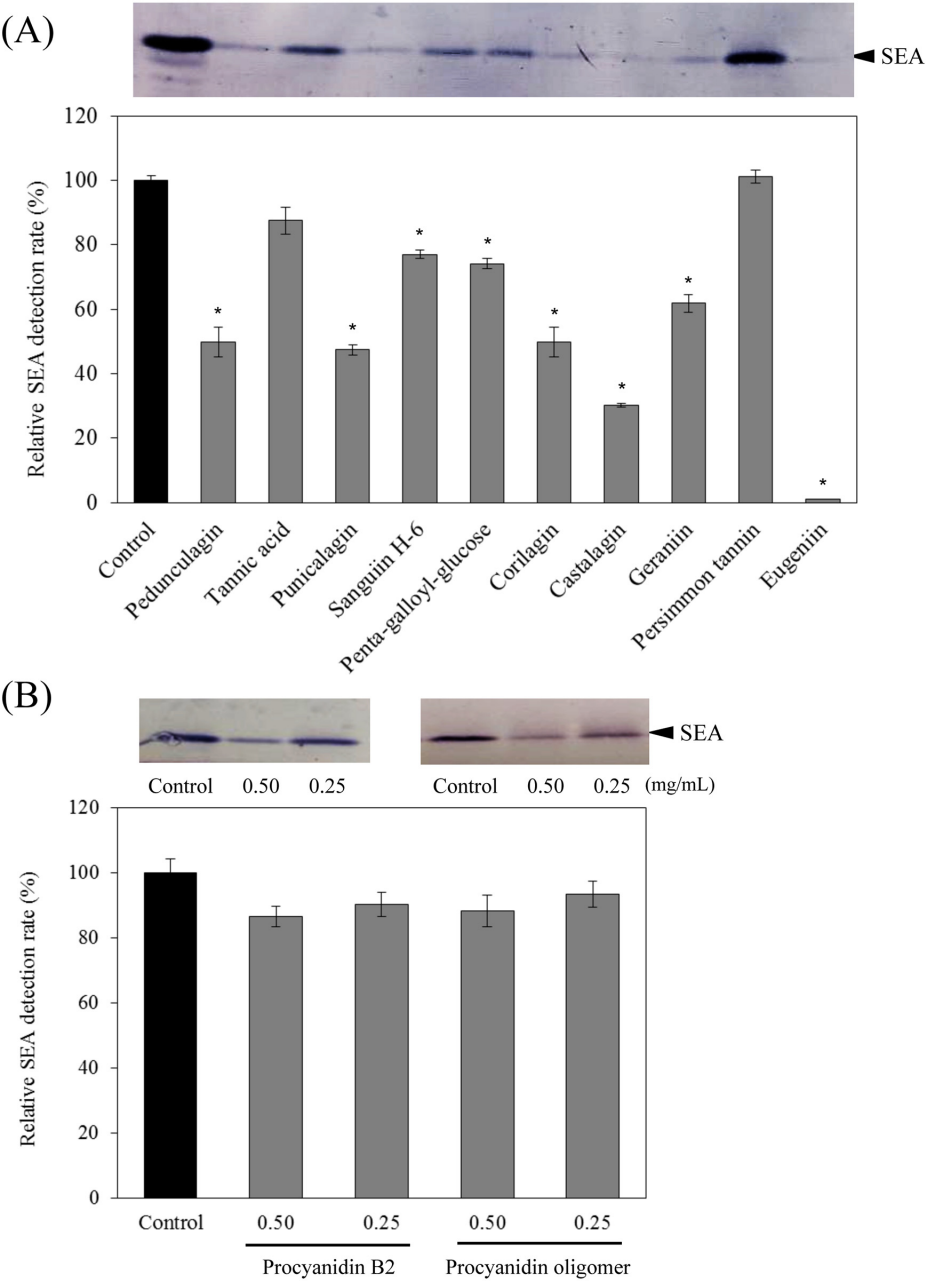
Direct reactivity of polyphenols to SEA. (A) hydrolyzable tannins (0.25 mg/mL) and (B) procyanidins (0.25–0.50 mg/mL). Values represent the mean ± SD for three independent experiments. * represents *p* < 0.05 compared with the control. (); final concentration (mg/mL).

Considering the possibility that 12 polyphenols directly react with SEA or inhibit the production of SEA, we examined whether these polyphenols could react with SEA itself. After incubating SEA with the polyphenols, the supernatant centrifuged from the reaction mixtures was separated by SDS-PAGE gels. The reactions of the anti-SEA antibody with the polyphenol-treated and the untreated SEA samples were detected by Western blot analysis with an anti-SEA antibody. The precipitates were not formed when SEA was incubated alone. However, when SEA and some polyphenols were incubated, a precipitate was generated in the reaction mixtures. Therefore, reaction of SEA with polyphenols was evaluated by observing the presence or absence of the SEA band in the supernatant after centrifugation. The SEA and each of 12 polyphenols (pedunculagin, tannic acid, punicalagin, sanguiin H-6, penta-galloyl-glucose, corilagin, castalagin, geraniin, persimmon tannin, eugeniin, procyanidin B2 and procyanidin oligomer) were mixed and incubated at 37°C for 24 h. After incubation, a precipitate was formed when SEA was incubated with 8 out of 12 polyphenols (pedunculagin, punicalagin, sanguiin H-6, penta-galloyl-glucose, corilagin, castalagin, geraniin and eugeniin). As a result, the SEA protein band was not detected in the supernatant by Western blot analysis (Fig 3(A) and 3(B)). It was suggested that the aggregates had formed in the reaction mixture from the interaction of SEA and these polyphenols. Of the 12 polyphenols, 8 polyphenols (0.25 mg/mL) significantly diminished the band intensity of the SEA protein (*p* < 0.05) (Fig 3(A)). These results indicate that 8 polyphenols may react with the SEA protein. As a result of the Western blot analysis, no significant difference was observed at the position of the SEA protein band when SEA was incubated with 8 polyphenols. It was reported that a higher ionic strength decreases the protein solubility after incubation with procyanidin B2 [25]. Verhelst et al. (2013) also demonstrated that the *Escherichia coli* heat-labile enterotoxin is precipitated by certain polyphenols [26]. Hydrophilic proteins are influenced by the surface properties of the molecules, decreasing the protein solubility [27]. Our result of the formation of precipitates when SEA and polyphenols were incubated together was similar to previous reports [25–27]. On the other hand, after incubation at 37°C for 24 h, precipitates were not observed for 4 out of the 12 polyphenols (tannic acid, persimmon tannin, procyanidin B2 and procyanidin oligomer), and most of the SEA protein band was detected in the supernatant by Western blot analysis (Fig 3(A) and 3(B)). Because these 4 polyphenols reduced the SEA protein band intensity when reacting with the SEA-producing strain (Fig 2(A) and 2(B)), they may not directly react with SEA protein. Instead, they may exert an inhibitory effect on the production of SEA in the SEA-producing strain.

### Inhibition of toxin activity

**Fig 4:**
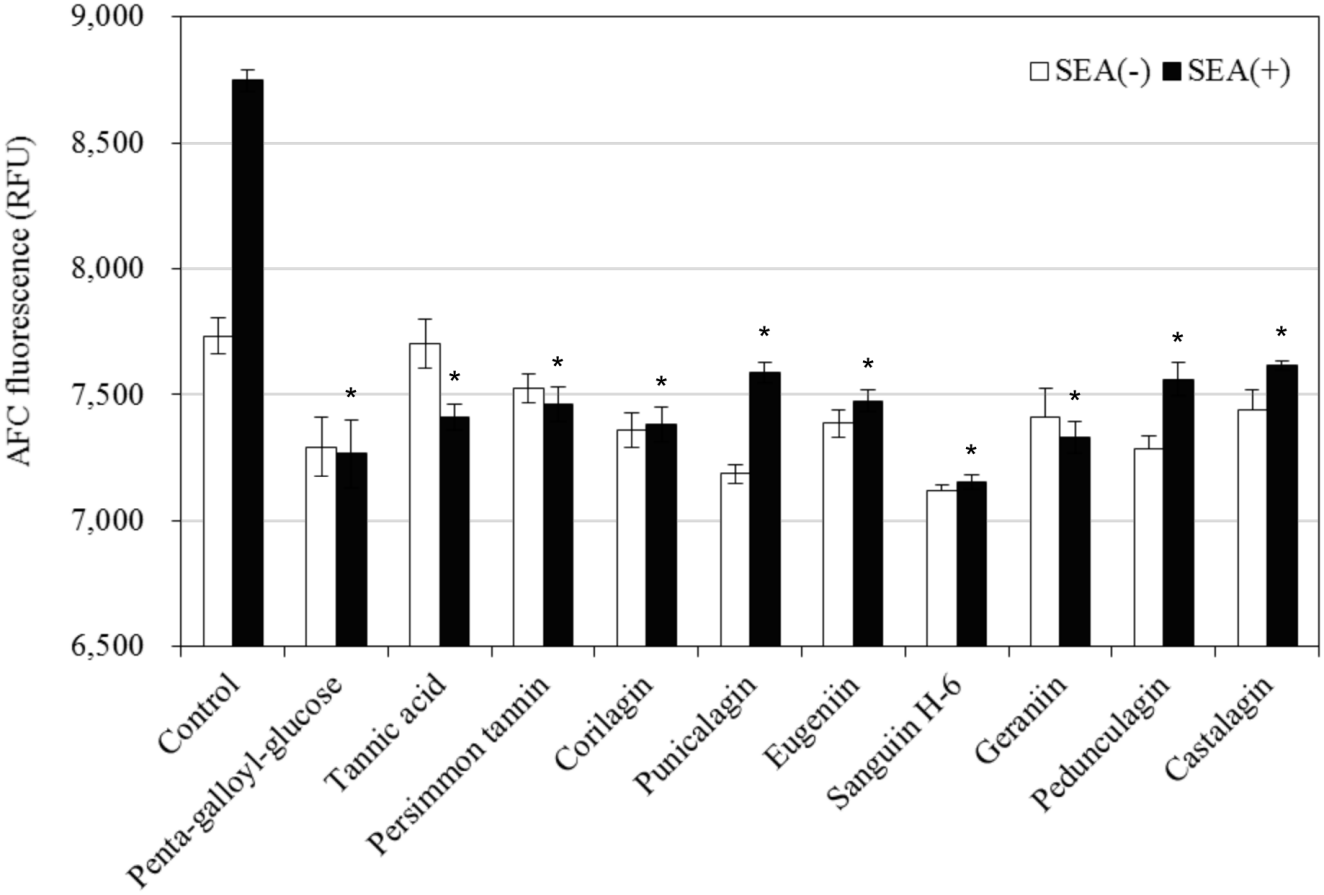
Inhibitory effect of polyphenols on the toxic activity of SEA against spleen cells. Values represent the mean ± SD for three independent experiments. * Represents *p* < 0.05 compared with the SEA (+) control. (); final concentration (μg/mL).

It was examined whether the 10 polyphenols with (8 polyphenols: penta-galloyl-glucose, corilagin, punicalagin, eugeniin, sanguiin H-6, geraniin, pedunculagin and castalagin) and without (2 polyphenols: tannic acid and persimmon tannin) SEA interaction suppress toxin activity. The concentration of each polyphenol that did not affect the spleen cells was used (penta-galloyl-glucose, 25 μg/mL; tannic acid, 12.5 μg/mL; persimmon tannin, 6 μg/mL; corilagin, 3 μg/mL; punicalagin, 3 μg/mL; eugeniin, 3 μg/mL; sanguiin H-6, 12.5 μg/mL; geraniin, 6 μg/mL; pedunculagin, 3 μg/mL and castalagin, 3 μg/mL). As a result, all of the 10 polyphenols inhibited GF-AFC cleavage by splenocyte proliferation induced by SEA (Fig 4, *p* < 0.05). These results indicate that these polyphenols inactivate the biological activity of SEA. SEA has both emetic and superantigenic activities. The SEA cross-bridge T-cell receptors (TCRs) have major histocompatibility complex class II (MHC II) molecules on antigen-presenting cells (APCs), inducing the proliferation of a large number of T-cells [28, 29]. Maina et al. demonstrated that SEA-induced cytokine production and emesis critically depend on the specific regions of the SEA molecule and residues 35–50 and 81–100 of the SEA. These regions could be important for the induction of both emetic and superantigenic activities [30]. If these polyphenols bind to specific regions of the SEA that are associated with the toxin active site, the emesis and superantigenic activities that are induced SEA may be inhibited. Therefore, it will be necessary to examine whether these polyphenols interact directly with the toxin active site of the SEA.

### The theoretical relationship between the polyphenol structure and inhibition of staphylococcal toxin activity

**Fig 5:**
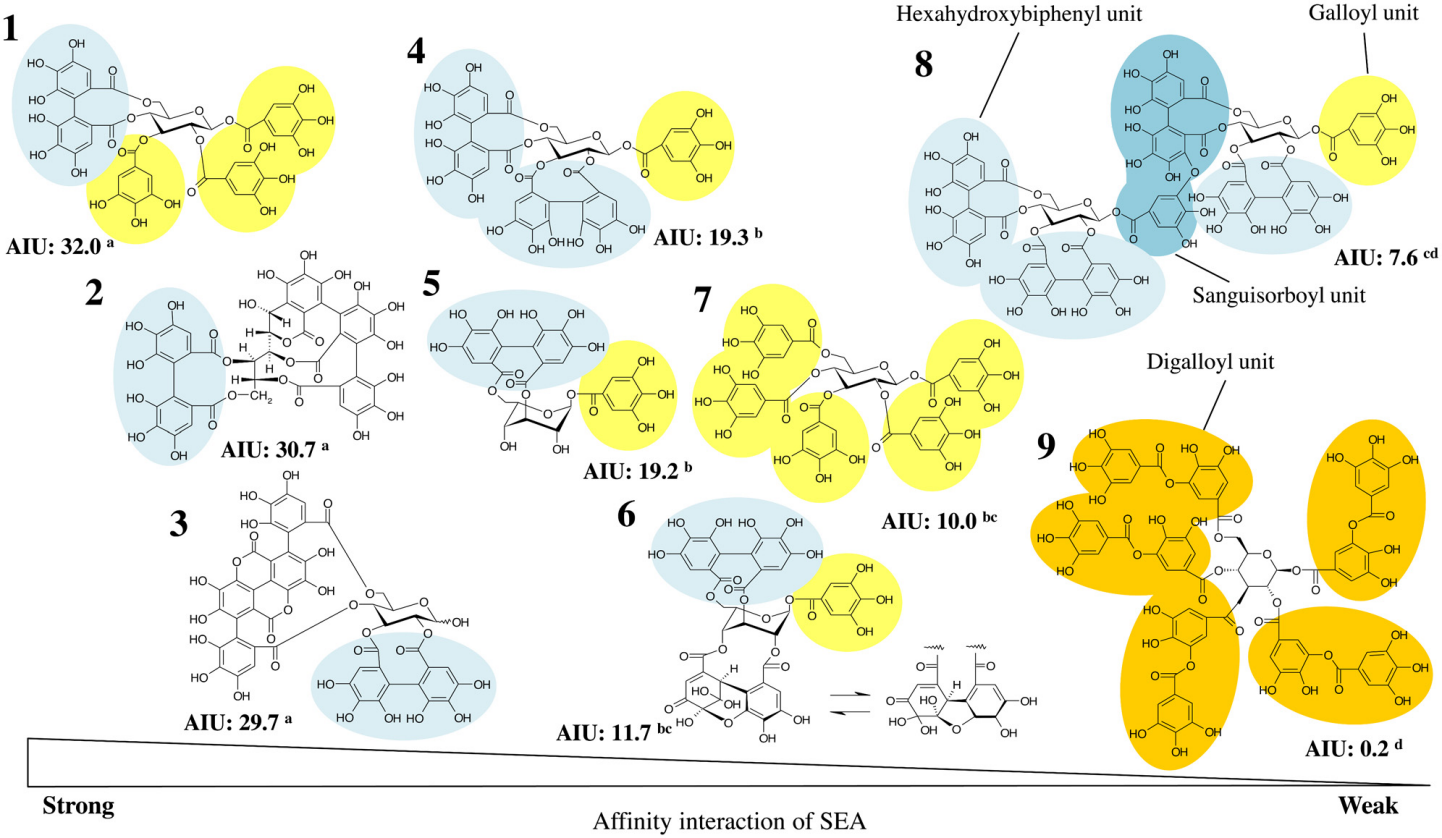
Affinity interaction of staphylococcal enterotoxin A (SEA) and hydrolyzable tannins. 1; eugeniin, 2; castalagin, 3; punicalagin, 4; pedunculagin, 5; corilagin, 6; geraniin, 7; penta-galloyl-glucose, 8; sanguiin H-6, and 9; tannic acid. For the definition affinity interaction unit (AIU), see the Materials and methods section.

We used Western blot analysis to examine whether the SEA protein band intensity, when SEA is treated with polyphenols, is correlated with the polyphenol structure. An affinity interaction unit (AIU) of polyphenols and SEA was calculated based on the degree of the band intensity of SEA treated with polyphenols by Western blotting analysis (Fig 5). The hexahydroxydiphenoyl (HHDP) group is a motif of ellagitannins, such as eugeniin, corilagin, castalagin, pedunculagin, punicalagin, geraniin and sanguiin H-6. It is a class of polyphenolic natural products with a broad range of biological activities [31–33]. Sanguiin H-6, which has three HHDP groups and one sanguisorboyl group, and tannic acid, which has a digalloyl group, had a weak affinity interaction with SEA. In addition, penta-galloyl-glucose, which has only one galloyl group and lacks HHDP groups, also showed a weak affinity interaction with SEA. These results suggest that polyphenols that have one or two HHDP groups and/or one galloyl group, such as eugeniin, castalagin, punicalagin, pedunculagin, corilagin and geraniin, exhibit a strong interaction with SEA. There was a significant difference between polyphenols with one or two HHDP groups and no HHDP groups when we performed the Tukey–Kramer method based on the SEA protein band intensity. The polyphenol structures contain electron-rich aromatic structures and ionizable phenolic OH groups. Rasooly and Friedman speculated that these polyphenol structures can have varying toxin activity via non-covalent binding to the SEA protein and/or by altering the distribution of ionic charges via H-bonding between OH groups and ionizable groups of the SEA protein [34]. Generally, it has been reported that hydrolysable tannins mainly form a hydrophobic coating on the protein surface [35], and polyphenols and proteins have a covalent interaction [36]. Additionally, it was confirmed that the phenolic group forms hydrogen bonds with the carboxyl group of the protein. In addition, polyphenols that have high protein affinity must be small enough to travel through protein molecules and large enough to crosslink peptide chains [37]. In this study, the molecular weight of the polyphenols that weakly interacted was more than 1000 (sanguiin H-6; 1871.3, tannic acid; 1701.2), whereas the molecular weight of the polyphenols that strongly interacted was 1000 or less. These results suggested that the polyphenol molecular weight was the important factor that affected the interaction between polyphenol and SEA. Although, we have no direct evidence for an actual interaction between various polyphenols and SEA or the binding mechanisms, the data in this manuscript are useful as the first screening of various compounds, such as polyphenols or plant components, that have reactivity with SEA. Additionally, this study evaluates the inhibitory effect on the toxic activity of SEA for preventing food poisoning with natural substances. However, the data presented here need to be confirmed in further studies to better understand the mechanism underlying the interaction between polyphenols and SEA and the inhibitory action of polyphenols on toxin activity induction.

## Conclusions

Some polyphenols interact with SEA and inhibit SEA-induced toxic activities without causing cytotoxicity. We examined whether the SEA Western blot analysis protein band intensity when SEA is treated with polyphenols is correlated with the polyphenol structure. As a result, polyphenols with one or two HHDP groups and/or one galloyl group, such as eugeniin, castalagin, punicalagin, pedunculagin, corilagin and geraniin, may have interacted with SEA and inhibited the toxin activity at a low concentration. Our findings suggest that polyphenols with these structures may be potentially useful ingredients for SEA inactivation. The polyphenols used in the present study interacted with SEA and had inhibitory effects on the SEA toxin activity without inhibiting staphylococcal growth. However, we have no direct evidence for reactions (covalent binding) and non-covalent binding between polyphenols and SEA. The inhibitory mechanism by which polyphenols act on SEA remains to be studied in future trials.

## Abbreviations

SEs: staphylococcal enterotoxins
SEA: staphylococcal enterotoxin A
BHI: brain–heart infusion
MIC: minimum inhibitory concentration
CFU: colony-forming unit
PVDF: polyvinylidene difluoride
BCIP/NBT: 5-bromo-4-chloro-3-indolyl phosphate and nitroblue tetrazolium
GF-AFC: glycyl-phenylalanyl-amino-fluorocoumarin
TCR: T-cell receptor
MHC: major histocompatibility complex
APCs: antigen-presenting cells
BAU: binding affinity unit
HHDP: hexahydroxydiphenoyl
EGCG: (−)-epigallocatechin gallate
